# The Height of Children and Adolescents in Colombia. A Review of More than Sixty Years of Anthropometric Studies, 1957–2020

**DOI:** 10.3390/ijerph18168868

**Published:** 2021-08-23

**Authors:** Adolfo Meisel-Roca, Angela Granger

**Affiliations:** Department of Economy, Universidad del Norte, Barranquilla 1569, Colombia; ameisel@uninorte.edu.co

**Keywords:** Colombia, height, regions, history, socioeconomic groups, ethnic group

## Abstract

In this article, we present a review of the studies on the heights of children and teenagers in Colombia published since 1957. We focus on examining the geographic coverage, features of the population studied, height measurement techniques, authors’ profiles, and growth patterns in children. This relatively recent literature has been developed mainly by medical doctors who carried out rigorous measurements with highly specific time and space horizons. The first studies emphasized the differences among socioeconomic levels. Later, there was an interest in minority groups, such as indigenous people and Afro-descendants. Although most of the research lacked long-term vision, the overall balance shows that the country has been improving in anthropometric indicators over time, across territories, and in different socioeconomic groups.

## 1. Introduction

Anthropometric indicators, especially height, have been extensively used in the health sciences and in economics to study biological well-being, both in a given moment and over time. The average values for children’s heights and weights reflect their nutritional status when the differences in genetic potential are properly taken into account [1]. For this reason, these variables are a good indicator of social progress, since they reflect the nutritional and health conditions during the years of growth: 0–21 years.

Growth in an individual occurs during childhood and adolescence. Part of it is the result of genetic inheritance but, also, a reflection of environmental or socioeconomic conditions [2]. The natural tendency towards balance between height and weight is one of the adaptation mechanisms to the environmental conditions of organisms [3]. For this reason, when comparing the growths of different populations (over time or crosswise), the differences in the environmental conditions to which they are subjected are indirectly examined. When the latter are unfavorable, the individuals do not reach the growth potential contained in their genes.

Beginning at the end of the 19th century, some doctors and anthropologists began to collect data on the height of the populations they studied, since they understood that anthropometric variables (height, weight, and body mass index) reflect the evolution of people’s physical conditions. For example, the famous North American anthropologist Franz Boas (1858–1942) collected measurements of the heights of school children in Toronto. As a result of his scientific interest, the average height of children in Canada at that time, and the fact that it rose ten centimeters between 1891 and 1974, are known [4].

In Latin America, studies of the heights of children first began in Chile in the 1890s. There, doctors who pioneered these measurements maintained their interest in the subject over time. For that reason, there is an abundant bibliography on the subject throughout the 20th century. From these studies, it is known that the average height of Chilean children and adolescents increases between 0.9 and 1.3 cm per decade, depending on their social class [5]. These results are very similar to those found in studies about the evolution of height in adults in the 20th century [6,7].

Compared to Chile, Colombia shows a lag in anthropometric studies, since it was only until the end of the 1950s that studies with observations on the heights of children and adolescents began to be published. These studies began to increase rapidly only at the beginning of the 21st century.

Another intellectual tradition from which interest in the study of height arose is that of economic historians in the United States. The use of anthropometric indicators in economic history gained strength in the late 1970s with the research of Robert W. Fogel. His interest in the subject arose from the debates surrounding his book “Time on the Cross: The Economics of American Negro Slavery” (1974) [8]. The author used information on the living conditions and nutritional status of slaves in the United States to challenge the abolitionists’ claims about malnutrition in that population. However, criticism of Fogel’s conclusions quickly emerged, because they did not account for the increased physical exertion that slaves went through, nor the unfavorable environmental conditions that made them prone to disease. This debate motivated an interest in compiling a greater number of figures that could fill in these gaps. In particular, Fogel and his associates collected data on adult heights, which is largely the result of nutrition, physical exertion, and environmental factors during growth, as well as genetics [9]. Since then, the use of height as an indicator of the evolution of the standard of living has spread throughout the world.

The aim of this article is to take stock of the studies that were produced on the heights of children and adolescents in Colombia, from the date of the first publication registered in 1957 to 2020. We review 37 publications in specialized medical or nutrition journals that, we believe, represent a large proportion of all the studies carried out in Colombia (we could find no additional articles on the topic for this period). We focus on geographic coverage, features of the population studied (at the ethnicity and socioeconomic levels), height measurement techniques, authors’ profiles, and growth patterns of children over the last sixty years. The results that emerge from the anthropometry of children in this balance coincide with those obtained from studying the heights of adults in sources such as passports, IDs, and criminal records. Both approaches coincide in a sustained increase in the average height in almost the entire territory and among different socioeconomic groups as a result of the improvements in nutrition and healthcare.

## 2. Methods 

These articles were compiled by seeking the widest possible coverage of studies on the heights of children and adolescents in Colombia. For this reason, instead of following a systematic search based on criteria that could leave certain studies out, we undertook an exhaustive search for articles with measurements of heights or stunting in Colombians between 0 and 19 years. As shown in Table A1, the studies were published between 1957 and 2020, with the data about children and young people born after the 1930s disaggregated by sex, age groups, and, usually, by socioeconomic level. Most of these studies were conducted in urban areas in the interior of the country with information collected from public and private schools.

## 3. Studies of the Height of Children and Adolescents in Colombia

The first reference that is known in Colombia about height in the context of health-related issues is from 1918, when, at the Third Colombian Medical Congress, Miguel Jiménez, MD presented estimates of the average heights of army recruits. According to Jiménez, they showed a large drop in average heights over time [10]. Supposedly, this information corroborated a degeneration of the race by miscegenation. His intention was racist, and his information was either manipulated to show what he wanted to demonstrate, or it corresponded to a nonrepresentative sample. We say this, because the empirical evidence from broad and systematic sources such as IDs and the criminal records show the opposite: a systematic and generalized increase in the heights of Colombians since the first decade of the 20th century (Further comments on this subject are made in references [11,12,13,14]). 

Apparently, it was only until 1957 that medical doctors were once again interested in the subject of heights in Colombia. That year, Julio Gómez, Antonio Úcros, and Gabriel Gómez, members of the Colombian Society of Endocrinology, physicians with the San José Hospital in Bogotá, studied the case of eleven siblings of a peasant family from Cimitarra, Santander [15]. This family had consulted the endocrinology service of the San José Hospital in 1955. They had a diet with an adequate caloric intake until 1948. From that year on, they had to emigrate from Cimitarra to Bucaramanga, due to growing political violence. Finally, they arrived in Bogotá in 1951. As unemployment affected the head of the household, all the children had a growing nutritional deficit, which led to chronic malnutrition. Until 1955, their caloric intake was insufficient, with a marked deficit of proteins and fats, given that they ate just a plate of corn soup twice a day. Likewise, the housing and personal hygiene conditions were completely inadequate during the same period. As a consequence, the siblings showed a marked deficit in their height.

The authors wrote that this case study allowed observations of the relationship between malnutrition and growth in two stages in which protein, fat, and supplementary requirements are absolutely necessary: in childhood and in preadolescence. As a result, when chronological age was compared with bone age in this family, the greatest morphological growth stagnation was observed in children between six and ten years of age (see Table 1). In these children, the prolonged episode of malnutrition affected the second growth spurt of childhood and adolescence [15].

**Figure 1:**
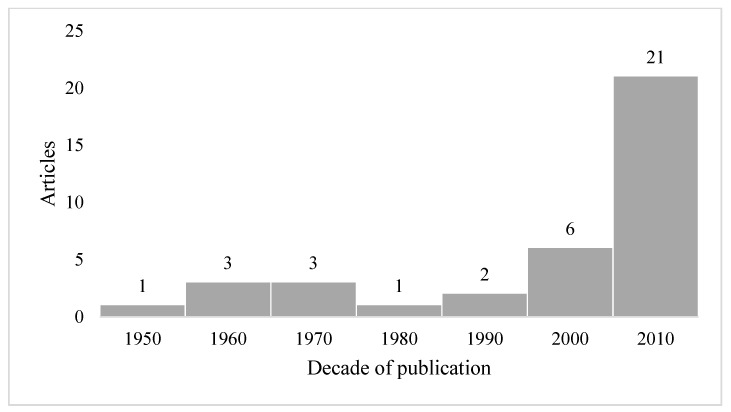
Articles on the heights of Colombian children and adolescents reviewed in this paper by decade of publication. Source: Created by the authors.

Interest in the study of growth and development in children and adolescents persisted in the years following this study, during which a series of investigations based on the collection of primary information in different cities and regions of Colombia was published. These studies began being published in the 1960s and grew over the years, until there was a wide proliferation in the first decade of the 21st century (see Figure 1). As shown in Figure 1, we reviewed a total of 37 articles, written between 1957 and 2020, on the heights of children and adolescents in the country.

We should highlight the reason why studying the heights of children and adolescents is so important from a medical point of view. The maximum height that an individual can reach depends on their genes and on the environmental factors that affect their physical growth between 0 and 21 years, which is the interval in which total height is usually reached. Environmental factors include adequate nutrition, health, and sanitary conditions, such as access to drinking water and sewerage.

Various features can be found in the 37 articles reviewed. The first is that, almost without exception, they are the product of research by physicians, often endocrinologists and pediatricians, and a few nutritionists. This research is heavily influenced by medical research conducted in the United States. For example, in the 1967 study carried out by Antonio Úcros et al., all six bibliographic references quote works published abroad.

The second characteristic of the bibliography on the heights of Colombian children and adolescents is that the authors usually compiled their own databases, which were obtained through careful measurements, many times in the educational institutions attended by the students selected in the sample (a detailed description of the measurement techniques is presented in Table A2). Therefore, the time horizons of these studies are usually limited, often cross-sections for one year, and their geographic coverage can also be very focused. In other words, they are rigorous studies from the point of view of the scientific method but without perspective on the long-term trends in heights or weights. Additionally, many of the studies referred only to Bogotá. Others included specific urban areas such as Medellín, Cali, Pereira, and Tunja. A few works were carried out in basically rural populations, such as San Jacinto-Bolívar, Mariquita-Tolima, and Marinilla-Antioquia. The first study with national coverage for the height of children and adolescents was carried out in 1982 by the National Institute of Health (Instituto Nacional de Salud, INS). (The National Nutrition Institute (NNI) was founded in 1947. In 1963, Doctor Roberto Rueda Williamson was appointed as the director. He promoted the strategic importance of the fight against malnutrition in children and adolescents. The NNI disappeared in 1968, since it was absorbed by the Colombian Institute of Family Welfare [16]).

A third characteristic is that these early studies focused heavily on observing and discussing differences in heights between different socioeconomic classes. One of the reasons for prioritizing this aspect of the analysis is that, at the time, the differences in heights according to social class were much more marked than today. For example, in the study by José Obdulio Mora in 1967 for San Jacinto, Bolívar, almost all of the children were of low or very low socioeconomic levels. Compared with members of the upper class of Bogota, the 17-year-old males were found to be 5.3 cm shorter in San Jacinto [3]. Another reason for concentrating on social class was that, having basically data for one year, these inequalities, at one point in time, were almost the only subject available for analysis and discussion.

Finally, a good number of studies refer to vulnerable groups, such as indigenous people and Afro-Colombians. In the case of indigenous people, in the research by Berta Nelly Restrepo et al. on the Embera-Katios of Tierralta, Córdoba in a sample of 272 children up to six months of age, it was found that 84.9% of the children had delayed heights [17]. This is very serious and even higher than the situation of other indigenous groups, such as the Arhuacos of the Sierra Nevada de Santa Marta, who had a prevalence of stunting in 51.2% of the children. 

Among the studies about vulnerable populations and the effects of malnutrition, we will mention that of Rubén Andrés Ortega et al., who studied the heights of children and adolescents between 5 and 18 years old in three municipalities of Cauca (Guapi, López de Micay, and Timbiquí) [18]. In that area, 95% of the population is of African descent. A sample of 6793 individuals was collected in the schools they attended. The percentage of schoolchildren with delayed heights observed was 13.9%, that was above the national average, which, in 2015, was 10.8%.

One of the limitations of several of the reviewed articles is that they are highly focused on medical and nutritional issues, without taking into account the central role of the social and economic variables that influence height in relation to age, such as access to drinking water. Another limitation of almost all of this literature published by physicians and nutritionists is that there is an excessive concentration in the samples of the local scope or of a very limited temporality, often with samples taken over a single year. This problem is not present in the tradition of the anthropometric studies that emerged in the second half of the 1970s in the United States with the works of Stanley L. Engerman and Robert W. Fogel [19]. From the beginning, anthropometric historians had a long-term approach and devoted themselves to studying major trends in the biological well-being of human beings. Their contributions have been very significant, and there is a growing bibliography on the subject [20]. 

In Colombia, a pioneering work, influenced by the anthropometric research of Fogel and his associates, was carried out in Fedesarrollo (Higher Education and Development Foundation) and published in 1992. Although it was carried out by two doctors, Antonio Ordoñez and Doris Polanía, the topic had been suggested to them by the director of that center, Miguel Urrutia, an economist who knew about Fogel’s work on heights. The authors used the information contained in the Colombia national ID and built a database with 14,103 observations taken in the period of 1910–1970. Their main conclusion was that, in this period, the heights of Colombians increased steadily, 8.7 cm for women and 7.0 for men [21]. Unfortunately, these authors stuck to a description of what the data showed and did not try to systematically explain what was observed through the determinants of height.

In 2007, Adolfo Meisel and Margarita Vega published an article and a book studying the evolution of the heights of Colombians between 1870 and 2003. For the period 1905–2003, they used data from the national ID, and from 1870 to 1905, data from passports. In other words, the time horizon and the number of observations were extended, and heights were correlated with other economic variables. The authors found that, between 1910 and 1984, the heights of women and men increased by 9 and 8 cm, respectively, and furthermore, the dispersion of heights fell for this same period, thus showing a process of departmental convergence in biological well-being [11].

## 4. Empirical Evidence

In this section, we review the articles written mainly by physicians and nutritionists in 1957–2020 on the heights of Colombian children and adolescents. Ucrós and others published the first empirical research, carried out with a large database, in a collaboration between the San José Hospital and the INN. The authors studied chronological age, bone age, height, weight, and other indicators of sexual maturation in a group of 1800 students of both sexes, between the ages of six and eighteen years and with similar socioeconomic conditions, enrolled in public schools in the municipality of Mariquita, Tolima. The indicators calculated were compared with those obtained by the INN for a group of children of high socioeconomic status in Bogotá. The results showed that Mariquita’s students matured satisfactorily, and the relationship between chronological age and bone age was superimposed on the maturation observed in the upper socioeconomic group of Bogotá [22].

In 1967, a somatometric investigation was undertaken with 12,138 individuals between birth and 20 years of age, men and women, from different social classes in the city of Bogotá (Somatometry is the measurement of the physical dimensions of the human body: weight, height, and body mass index). The results of this research were published in 1971 by several professors from the School of Medicine of the National University (Universidad Nacional) in collaboration with the INN. The interest of the authors focused on comparing the growth of groups under different ecological conditions (referring to socioeconomic levels). The results expressed in height and weight percentiles for each age group; sex; and socioeconomic class (very low, low, medium, and high) indicate that the alterations in the direction and speed of growth are associated with adverse environmental conditions. In addition, the authors argued that most of the reference standards used at the time had been estimated, in general, on highly privileged socioeconomic groups and were not representative of the potential variability of growth of the population aggregate [2].

Ariza corroborated these differences between social classes later in an investigation where Colombian children from the highest socioeconomic classes achieved growth similar to the North American standard during the first year of life but were slightly lower in the following years. In contrast, children from the lowest socioeconomic levels were below the American standard and the upper-class average from birth [23].

Although the studies of these years referred mainly to Bogotá and the center of the country, this group of physicians from the INN also undertook an analysis of the growth of 766 low-income preschoolers in San Jacinto, Bolívar. This analysis was supported by a preliminary study of this population by the physician José Obdulio Mora in 1967. The children examined were residents of a locality with poor sanitation conditions and a high rate of intestinal parasitism. Mora found a delay in height and weight with respect to the standards established by the INN. The authors noted that:

*“Weight begins to be deficient after 12 months, and its delay is accentuated between 30 and 36 months; later, it shows a tendency to recover, which is much more evident when compared to what would be ideal for the height. On the contrary, the latter shows a marked deterioration after 18 months and its recovery is minimal [24]”.*

As mentioned, during their growth process, children present alterations in the weight–height relationship that essentially depend on the interaction between nutrition and the natural tendency to balance as a mechanism of adaptation of the organism to adverse environmental conditions. Low weight is a reflection of current malnutrition, and a decline in height is a manifestation of chronic malnutrition [3]. This tendency to balance explains much of the results of these studies in which the weight-for-height indicators adjusted over time but the weight-for-age did not.

In the following decades, the literature in this area was fed with information from children and adolescents from different regions of Colombia, mainly from the center of the country: in Aburrá Valley [25], in Medellín [26,27] and other municipalities of Antioquia [28,29,30,31], in Bogotá [32,33], Tunja [34], Pereira [35], other main cities such as Barranquilla and Cali [36] and municipalities such as La Mesa, Cundinamarca [37], and Puerto Colombia, Atlántico [38].

In 1992, José Mora and others used data from the National Health Study, an anthropometric survey in the periods of 1965 to 1966, 1977–1989, and 1986–1989, to study the evolution of the nutritional conditions of children in Colombia between 1965 and 1989. The authors found a decrease in the prevalence of malnutrition of 52% and a drop of 48% in the prevalence of stunting in children between 0 and 59 months of age. As Mora et al. argued, this transformation was the result of social progress in Colombia during the second half of the 20th century [39]. These were years of growth in public spending, especially in infrastructure (electrical networks, roads, and communications); public services (aqueducts and sewers); and social services [40]. 

Parallel to the bibliography on height developed by doctors and nutritionists, literature on the subject also emerged in Colombia with an emphasis on the evolution of the biological quality of life in the long term. A prime example of this is the study by Ordoñez and Polanía [21]. As noted, this type of research was inspired by the work of the economic historians of the cliometrics school.

In the 21st century, national surveys have begun to be used for the analysis of anthropometric indicators of children. The most widely used have been the National Demographic and Health Survey (Encuesta Nacional de Demografía y Salud, ENDS) carried out by Profamilia every five years since 1990 and the National Survey of Nutritional Situation (Encuesta Nacional de Situación Nutricional, ENSIN), which has been carried out every five years since 2005 by ICBF, the Ministry of Health and Social Protection, the Administrative Department for Social Prosperity (Departamento Administrativo para la Prosperidad Social, DPS), INS, and the Pan American Health Organization (PAHO). For example, Gaviria and Palau studied the incidence and determinants of child malnutrition in Colombia with data from the 2005 ENDS [41]. García et al. used the same survey to examine the socioeconomic inequalities in malnutrition among Colombian children and adolescents and to evaluate the contribution of individual, family, and community factors to these inequalities [42]. Parra et al. estimated the prevalence of malnutrition and obesity at the individual and family levels in five-year-old children, schoolchildren, adolescents, and adults with information from the 2010 ENDS [43]. Amarante, Figueroa, and Ullman focused on the evolution of child growth delay in seven Latin American countries between 2000 and 2010. In the case of Colombia, they used the information from the ENDS and the ENSIN published for those periods [44].

In addition to the implementation and use of national surveys, there has been a marked concern in this century for the analysis of the growth, development, and nutrition of children of ethnic minorities, particularly from indigenous and Afro-Colombian populations. Restrepo et al. estimated the prevalence of malnutrition in 272 Embera-Katío indigenous boys and girls aged 0–6 years in Tierralta, Córdoba between 2001 and 2002 and identified the factors associated with their nutritional status. The authors calculated the height-for-age, weight-for-height, and head circumference and contrasted them with the reference values of the United States National Center for Health Statistics, recommended by the World Health Organization (WHO). Children who had less than the median height-for-age by one standard deviation (SD) were classified with mild chronic malnutrition, those two SD below were classified with moderate chronic malnutrition, and those three SD below with severe chronic malnutrition. The results indicated a prevalence of chronic and moderate malnutrition of 63.4%, which was almost five times higher than the national average in 2000 and higher than that of other indigenous groups in Colombia and Latin America [17]. These results are related to the accumulated deficiencies of a diet low in protein and micronutrients and a high vulnerability to infectious diseases.

Likewise, Rosique et al. studied the nutritional status and anthropometric indicators of indigenous Embera in the municipality of Frontino, Antioquia [45]. The results were very similar to those of Restrepo et al.: chronic malnutrition in 68.9% of the children between zero and ten years old and short heights in 77.1% of young people between ten and nineteen years old. Arias et al. studied the case of the Arhuaco indigenous group, and again, the results showed high rates of malnutrition and stunting compared to the national averages and to other indigenous groups in Latin America [46]. Vallejo-Solarte et al., on the other hand, showed malnutrition and stunting in approximately 50% of the children from 0 to 5 years of age in the community of the Yunguillo Reservation in the municipality of Mocoa, Putumayo [47]. These results reflect the nutritional vulnerability of the indigenous population in Colombia and the lack of progress over time. This is also the case of people in many rural areas [48].

Ortega, Basto, and Chito used information for 6793 schoolchildren between 5 and 18 years of age, compiled by the health survey of the School Food and Nutrition Program (Programa de Alimentación y Nutrición Escolar, PANES) in the department of Cauca in an area where 96.1% of the population is of African descent. The levels of stunting and overweightness (13.9% and 12.2%) were lower than the national average for children and adolescents in the Colombian Pacific (16.3% and 13.2%), while the prevalence of thinness and obesity was 25% higher than that reported by the 2010 ENSIN [20]. Acosta and Meisel analyzed the evolution of the heights of Colombians of different ethnicities born between 1965 and 1990 [49]. They found that Afro-Colombians were the tallest group, which is consistent with the findings of Ortega, Basto, and Chito. Additionally, Acosta argued that the nutritional status of children in the Colombian Pacific, compared to those in other regions of the country, showed a notorious lag. According to the author, the departments of Cauca and Nariño (without the coast) and the Pacific coast registered, in 2010, percentages of malnutrition similar to those estimated for Colombia at the beginning of the 1990s [49].

Interdisciplinarity has become more recurrent in research in recent years. The literature that, in the previous century, had been exclusively authored by physicians, nutritionists, and other professionals in the health sciences was complemented by the works of statisticians, mathematicians, and economists [27,41,50,51,52]. The increasing availability of information was used to contrast the results with the North American or WHO standards but, also, the evolution of these indicators with other countries in the region and the world.

Studies on the growth and development of children and adolescents in Colombia published between 1957 and 2018 also showed a different perspective on the evolution of the standard of living compared to what was usual among Colombian economic historians until very recently. The empirical literature reviewed for this document was mainly published in journals related to health sciences, such as the *Latin American Archives of Nutrition*; *Biomédica*; *Public Health Nutrition*; and journals of the medical schools of different Colombian universities (Universidad del Norte, Universidad del Rosario, Universidad Nacional de Colombia, CES, and others) (see Table A3), and has not been referenced by economists and other social scientists.

Finally, it is possible to contrast the data available in some of these articles to show the evolution of the main anthropometric indicators in Colombia in the last fifty years. For this, we took the figures from two articles that contained information on the weight, height, and body mass index for children and young people between 0 and 17 years of age born between 1950 and 2010. The first was a study by Luna-Jaspe et al. with a sample of 12,138 individuals of all socioeconomic levels in Bogotá at the end of the 1960s [2]. The second, which offered more recent data, was that of Durán et al., in which they took anthropometric measurements of 27,209 children of medium and high socioeconomic levels in the four main cities of Colombia, Barranquilla, Bogotá, Cali, and Medellín between June 2009 and November 2010 [36]. Contrasting the indicators of these two studies is illustrative of the social change that has been reflected in the anthropometric indicators of children and adolescents since the second half of the 20th century.

**Figure 2:**
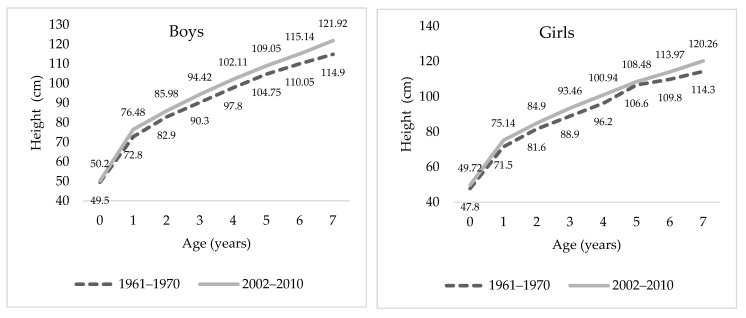
Median height evolution between children aged 0–7 years by sex. Sources: Authors’ calculations based on Luna–Jaspe et al. [2] and Durán et al. [36].

As shown in Figure 2, the difference in heights between newborn boys in the 1960s and in the 2000s is about 0.7 cm. Among children aged three to five, the difference between the two cohorts is approximately 4.2 cm, and by age seven, it is 7 cm. Among girls, the evolution of height is very similar to that of boys. The differences between the two cohorts were 1.9 cm at birth, approximately 4 cm between three and five years, and 6 cm at seven years (see Figure 2).

**Figure 3:**
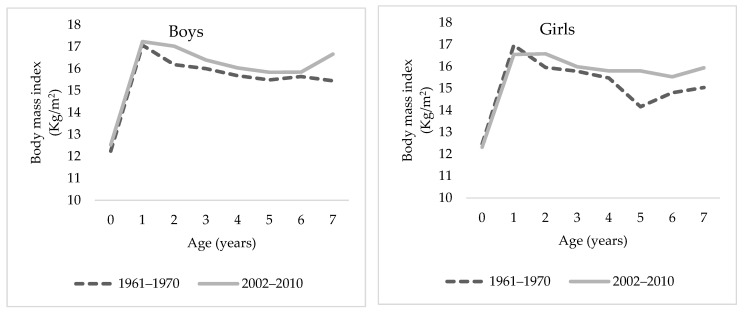
Median BMI evolution between children aged 0–7 years by sex. Sources: Authors’ calculations based on on Luna–Jaspe et al. [2] and Durán et al. [36].

As to the body mass index (BMI), a growth of 12 kg/m^2^ to approximately 16 kg/m^2^ is observed during the first year of life in both sexes, and from there, a decrease begins until the age of seven, which was slightly more pronounced in those born in the 1960s than in those born in the first decade of the 21st century. Despite this, the differences between the cohorts were, in general, less than 1 kg/m^2^ (see Figure 3), and, as expected, the body mass index was higher in men than in women.

**Figure 4:**
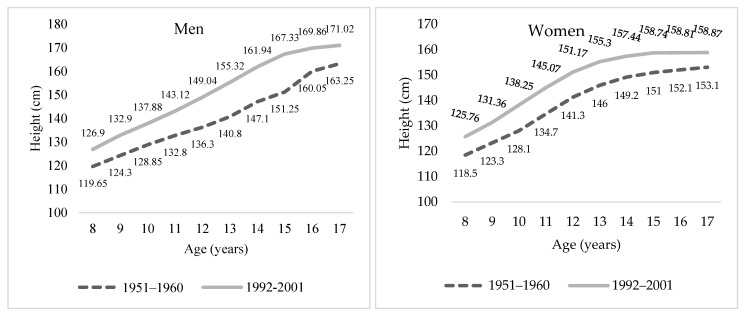
Median height evolution between children aged 8–17 years by sex. Sources: Authors’ calculations based on on Luna–Jaspe et al. [2] and Durán et al. [36].

As can be seen in Figure 4, the differences between the sexes were found at the most advanced ages. The heights of boys between 8 and 17 years old born in the 1990s were approximately 11 cm greater than those born in the 1950s. In contrast, in girls, there was a difference of up to 10.3 cm for the ages from 10 to 12 years old and 6 cm for girls between 16 and 17 years old. This is related, on the one hand, to gender differences in prepubertal maturation ages: the “hormonal surge” happens earlier in females than in males. On the other hand, nutrition is also important, as it impacts pubertal development. Better nutrition usually implies greater pubertal development. Additionally, this contrast supports the results, seen in other studies, of a generalized growth in the heights of Colombians, especially during the second half of the 20th century.

**Figure 5:**
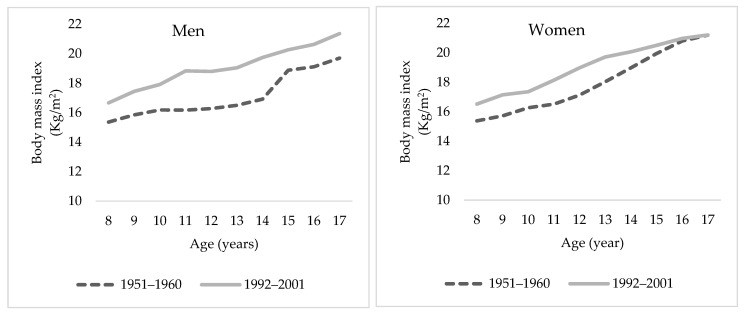
Median BMI evolution between children aged 8–17 years by sex. Sources: Authors’ calculations based on onLuna–Jaspe et al. [2] and Durán et al. [36].

Regarding to the evolution of the body mass index among young people aged 8 to 17 years, the difference between those born in the 1950s and 1990s were approximately 2 kg/m^2^ in males and 1.06 kg/m^2^ in females (see Figure 5). This BMI trend is similar to that found by Buyken et al. in a study of the evolution of BMI from childhood to adolescence in Germany. The authors found a BMI of around 16 kg/m^2^ in 8-year-old children and 21 kg/m^2^ at 17 years of age, with small differences between males and females (less than 1 kg/m^2^) [53].

## 5. Conclusions

The final height of an individual, which is reached around 21 years of age, is the result of the genetic potential with which they are born and the living conditions during their first years of life, especially the first three years. Reaching their potential height will depend mainly on the nutrition and health conditions in which children and adolescents grow. Achieving the genetic potential of their height is very important, since it is related to their productive capacity, intellectual development, and health throughout life. In countries with high levels of chronic malnutrition, a good part of the population is not fully productive in adulthood. A World Bank study by Emanuela Galasso and Adam Wagstaff found that, in terms of per capita gross domestic product, the economic cost for Colombia of the delay in population heights is between 3% and 5% [54]. In addition, these authors calculated that the profitability of nutrition programs aimed at eliminating the prevalence of stunting according to age is about 12%, a return hardly observable in infrastructure investment programs.

In sum, the studies published in Colombia on the delayed physical growth in children and adolescents show that the country has been progressing steadily over time across the territory and in every social class. These results are consistent with the work of Adolfo Meisel and Margarita Vega, who found a convergence in the height of Colombians by region and social class [11]. The exceptions are some social groups affected by violence and ethnic exclusion, such as the indigenous people of the Embera-Katío group, 84.4% of whose children were short for their age in 2006. 

The main limitation of this study was the relatively late development of the literature on height growth of the children and adolescents in Colombia. This made it difficult to assess long-term patterns, as has been done for other countries.

The advantage of studying the anthropometric measures of adolescents and children is that it allows health authorities to detect malnutrition problems and their effects in order to act and solve problems before it is too late, because, as we know, by the age of 21, almost all individuals reach their maximum height. For all these reasons, we conclude that indicators of children’s and adolescents’ weights and heights need to be monitored continuously. 

## Figures and Tables

**Table 1:** Sizes and ages of a family displaced by political violence.

Name	Chronological Age	Morphological Age	Bone Age
Isabel	17.8	11.5	15.0
Alicia	16.3	8.5	11.0
Manuel	14.0	12.0	12.6
Elvira	13.1	8.5	12.0
Beatriz	12.5	6.0	-
Reinaldo	10.0	3.5	3.0
Octavio	10.0	2.6	28.0
Rafael	7.3	3.0	2.8
Ricado	4.1	2.0	1.6
Hortensia	4.1	1.5	2.1
Pablo	1.1	2.0	-

Sources: Authors’ calculations, based on Ucrós, Gómez, and Gómez [15].

## Data Availability

No new data were created or analyzed in this study. Data sharing is not applicable to this article.

## Appendix A

**Table A1:** Description of the 37 studies about the heights of children and adolescents in Colombia, 1957–2020.

No	Title	Publication Year	Survey Year	Birth Cohorts	Ages	Sample Size and Sex	Sample Location	Urban–Rural	Sample Description
1	La nutrición como un factor extrínseco del desarrollo humano	1957	1955	1937–1953	1–17	F(5) M(6)	Bogotá	Urban	Eleven children from a low income family
2	Crecimiento y desarrollo	1967	1967	1948–1961	6–19	F(900) M(900)	Mariquita, Tolima	N.A	Public school students
3	Análisis del peso y la talla en 2.980 observaciones. San Jacinto (Bolívar), Colombia, 1967	1969	1967	1950–1967	0.5–17	F(1590) M(1390)	San Jacinto, Bolívar	Rural	Very low, low, medium, and high socioeconomic levels
4	El peso y la talla al nacimiento en un grupo de niños de clase económica baja. Manizales, Colombia	1969	1965–1966	1965–1966	0	870 (both sexes)	Manizales	Urban	Very low socioeconomic level
5	Somatometria en niños de clase socioeconómica baja—San Jacinto	1970	1967	1960–1967	0.5–6	F(375) M(391)	San Jacinto, Bolívar	Rural	Very low, low, medium, and high socioeconomic levels
6	Estudio seccional de crecimiento, desarrollo y nutrición en 12.138 niños de Bogotá, Colombia	1971	1969	1949–1969	0–20	F(6245) M(5893)	Bogotá	Urban	Medium and high socioeconomic levels
7	A cross sectional study of growth of Colombian children from two socioeconomic classes, during their first six years of life	1973	1969	1949–1969	0–6	F(1258) M(1238)	Bogotá	Urban	Very low and high socioeconomic levels
8	El adolescente en Colombia: Variables fisiológicas del desarrollo Pondo—Estatural y Sexual [55]	1982	1968–1975	1957–1962	6–18	2500 (ambos sexos)	N.A	N.A	Medium and high socioeconomic levels
9	Estudio longitudinal de crecimiento y desarrollo general en grupo de individuos del Valle de Aburrá	1991	N.A	N.A	8–18	F(608) M(607)	Valle de aburrá, Antioquia	Urban	N.A
10	Consistent Improvement in the Nutritional Status of Colombian Children between 1965 and 1989	1992	1965–1989	1960–1989	0–5	4683 (ambos sexos)	Nacional	Urban	All socioeconomic levels
11	Cultura Somática de adolescentes de 14–17 años escolarizados en Medellín: Características antropométricas	2002	N.A	N.A	14–17	F(179) M(157)	Medellín, Antioquia	Urban	Students from public and private schools of all socioeconomic levels
12	Velocidad media de ganancia de peso y estatura en niños de 2 a 10 años pertenecientes a familias del área rural del municipio de marinilla-Antioquia, Colombia	2004	N.A	N.A	2–10	F(122) M(137)	Marinilla, Antioquia	Rural	Vegetable producing families with children
13	Estado nutricional de niños y niñas indígenas de hasta seis años de edad en el resguardo Embera-Katío, Tierralta, Córdoba, Colombia	2006	2001–2002	1995–2002	0–6	F(129) M(143)	Tierralta, Córdoba	Rural	Communities of the Embera-Katio reservation
14	Nutrición y salud infantil en Colombia: determinantes y alternativas de política	2006	2005	2000–2005	0–5	F(4511) M(4511)	Nacional	Urban-rural	All socioeconomic levels
15	Estado nutricional y condiciones de vida de los niños menores de cinco años del área urbana del municipio de Turbo, Antioquia, Colombia, 2004	2008	2004	1999–2004	0–5	F(318) M(288)	Turbo, Antioquia	Urban	Very low and low socioeconomic levels
16	Estado nutricional de niños de Antioquia, Colombia, según dos sistemas de referencia	2009	2006	2001–2006	0–5	F(1091) M(1199)	Antioquia	N.A	Very low and low socioeconomic levels
17	Stunting associated with poor socioeconomic and maternal nutrition status and respiratory morbidity in Colombian schoolchildren	2010	2006	1994–2001	5–12	F(1596) M(1504)	Bogotá	Urban	Low and medium socioeconomic levels
18	Estado nutricional y hábitos alimentarios en Indígenas Embera de colombia	2010	N.A	N.A	0–6	F(184) M(169)	Frontino, Antioquia	Rural	Embera oibida indigenous community from Atausí and Embera eyabida from Nusidó
19	Propuesta metodológica para la comparación de mediciones antropométricas entre una población base y una población objeto: una aplicación entre población colombiana y estadounidense	2011	COL (1987 a 1993)–USA (1988–1994)	1971–1986	7–16	10285 (ambos sexos)	8 ciudades capitales	Urban	School students
20	Factors associated with stunted growth in children below 11 years of age in Antioquia, Colombia, 2004	2011	2004	1993–2004	0–11	F(1465) M(1502)	Medellín, Antioquia	Urban	N.A
21	Variabilidad del peso, la estatura y el índice de masa corporal según desarrollo puberal y tipo de colegio en adolescentes de Medellín, Colombia	2012	1998–1999	1980–1990	10–18	F(828) M(477)	Medellín, Antioquia	Urban	Students of public and private schools
22	The double burden of malnutrition and its risk factors in school children in Tunja	2012	2010	1991–2005	5–19	F(603) M(565)	Tunja, Boyacá	Urban-rural	Students from public and private schools of all socioeconomic levels
23	Estudio transversal de crecimiento de los escolares bogotanos: valores de estatura, peso e índice de masa corporal de los siete a los dieciocho años	2012	N.A	N.A	7–17	F(23277) M(22406)	Bogotá	Urban	Students from public schools
24	Socioeconomic inequalities in malnutrition among children and adolescents in Colombia: the role of individual-, household- and community-level characteristics	2013	2005	1987–2005	0–17	30779 (ambos sexos)	Nacional	Urban	All socioeconomic levels
25	Estado nutricional y determinantes sociales asociados en niños Arahuacos menores de 5 años de edad	2013	N.A	2005–2011	0–5	F(68) M(86)	Sierra Nevada de Santa Marta	Rural	Aruhaca community
26	Estado nutricional antropométrico de los niños y adolescentes de 17 escuelas del área rural del municipio de La Mesa, Cundinamarca, Colombia, 2012	2014	2012	1996–2007	5–16	F(191) M(220)	La Mesa, Cundinamarca	Rural	School students
27	Determinantes socioeconómicos, inseguridad alimentaria y desnutrición crónica en población desplazada de primera infancia, Pereira, Colombia	2014	2013	2008–2013	0–5	F(31) M(35)	Pereira	Urban	Community of “Las Colonias” in Pereira.
28	Valoración del estado nutricional de la población escolar del municipio de Argelia, Colombia	2014	2009	1990–2004	5–19	F (742) M(786)	Argelia, Valle del Cauca	rural	School students
29	Determinantes sociodemográficos de la nutrición infantil en Colombia	2015	2010	2005–2010	0–5	F(5325) M(5721)	Nacional	Urban-rural	All socioeconomic levels
30	The nutrition transition in Colombia over a decade: a novel household classification system of anthropometric measures	2015	2000–2010	1995–2010	0–5	28823 (ambos sexos)	Nacional	Urban-rural	All socioeconomic levels
31	Colombian reference growth curves for height, weight, body mass index and head circumference	2016	2009–2010	1989–2010	0–20	F(13877) M(13332)	Bogotá, Medellín, Cali y Barranquilla	Urban	Medium and high socioeconomic levels
32	Características nutricionales de los escolares afrodescendientes de la costa pacífica Colombiana	2016	2014	1996–2009	5–18	F(3182) M(3611)	Guapi, Lopez de Micay y Timbiquí, Cauca	Rural	School students
33	Nivel y estado nutricional en niños y adolescentes de Bogotá, Colombia.	2016	2014–2015	1997–2006	9–17	F(3873) M(2806)	Bogotá	Urban	Students from public schools
34	Estado nutricional y determinantes sociales en niños entre 0 y 5 años de la comunidad de Yunguillo y de Red Unidos, Mocoa—Colombia	2016	2014	2009–2014	0–5	241 (ambos sexos)	Mocoa, Putumayo	Urban-rural	Inga community of the Yunguillo reservation
35	Inequalities in the reduction of child stunting over time in Latin America: evidence from the DHS 2000–2010	2018	2005–2010	2000–2010	0–5	27573 (ambos sexos)	Nacional	Urban	All socioeconomic levels
36	Peso, estatura e índice de masa corporal de niños y adolescentes de moderada altitud de Colombia	2018	2013	1995–2007	6–17	F(1159) M(1082)	Bogotá	Urban	Students from medium socioeconomic level
37	Un estudio sobre el crecimiento, estado nutricional y composición corporal en menores de quince años de Salgar, Puerto Colombia: variabilidad y determinantes sociales	2020	2019	2004–2018	0–14	F (104) M(92)	Puerto Colombia, Atlántico	Urban	School students

Source: Authors’ compilation. Note: Information not available are marked as NA.

**Table A2:** Height measurement techniques.

No	Study	Publication Year	Height Measurement Techniques
1	La nutrición como un factor extrínseco del desarrollo humano	1957	N.A
2	Crecimiento y desarrollo	1967	N.A
3	Análisis del peso y la talla en 2.980 observaciones. San Jacinto (Bolívar), Colombia, 1967	1969	Wooden infantometers were used for children under 3 years of age and rubber tape measures fixed on vertical surfaces, with wooden squares as upper stops, for those over three years of age.
4	El peso y la talla al nacimiento en un grupo de niños de clase económica baja. Manizales, Colombia	1969	An infantometer with slide clamp was used, with a fixed square ruler of approximation to the millimeter. The height measurement was repeated at 36 h.
5	Somatometria en niños de clase socioeconómica baja—San Jacinto	1970	N.A
6	Estudio seccional de crecimiento, desarrollo y nutrición en 12.138 niños de Bogotá, Colombia	1971	The size was taken without shoes and determined by means of a stadiometer and an infantometer with slide clamp and wood squares and metal measuring tapes of approximation to the millimeter.
7	A cross sectional study of growth of Colombian children from two socioeconomic classes, during their first six years of life	1973	Reclining length was measured in children under two years of age, without shoes and naked, by means of an infantometer with slide clamp. Older children were measured without shoes using height measuring devices similar to the infantometers and with a l-mm interval scale.
8	El adolescente en Colombia: Variables fisiológicas del desarrollo Pondo—Estatural y Sexual	1982	The subject had to be barefoot, with his back to a rigid tape measure, with the heels, buttocks, cervicodorsal column, and occiput tangential to it. The measurement was made by means of an element perpendicular to the tape measure on the most prominent part of the skull; arms down and shoulders in a natural position.
9	Estudio longitudinal de crecimiento y desarrollo general en grupo de individuos del Valle de Aburrá	1991	It was measured with a wooden height rod
10	Consistent Improvement in the Nutritional Status of Colombian Children between 1965 and 1989	1992	It was measured with a wooden height rod
11	Cultura Somática de adolescentes de 14–17 años escolarizados en Medellín: Características antropométricas	2002	Anthropometric measurements, taken by trained interviewers of the national health surveys following standard procedures, included height (after two years) to the nearest millimeter.
12	Velocidad media de ganancia de peso y estatura en niños de 2 a 10 años pertenecientes a familias del área rural del municipio de marinilla-Antioquia, Colombia	2004	It was taken by Nutritionists with previous training and standardization with a height rod of 1 mm of sensitivity.
13	Estado nutricional de niños y niñas indígenas de hasta seis años de edad en el resguardo Embera-Katío, Tierralta, Córdoba, Colombia	2006	Length and height were measured with an infantometer and portable stadiometer, to the nearest millimeter.
14	Nutrición y salud infantil en Colombia: determinantes y alternativas de política	2006	Colombian Demographic and Health Survey
15	Estado nutricional y condiciones de vida de los niños menores de cinco años del área urbana del municipio de Turbo, Antioquia, Colombia, 2004	2008	The height was taken in supine decubitus in children under two years of age with a wooden infantometer with a sensitivity of 1 mm. In those older than two years, height was taken standing up with a SECA bodymeter 208 stadiometer with a sensitivity of 1 mm.
16	Estado nutricional de niños de Antioquia, Colombia, según dos sistemas de referencia	2009	To measure length in children under 24 months an infantometer was used, which had a capacity of 1 m and a sensibility of 0.1 cm. For those with 24 or more months measurement was done using a stadiometer of the SECA brand (SECA, Deutschland) with a capacity of 2 m and a sensibility of 0.1 cm.
17	Stunting associated with poor socioeconomic and maternal nutrition status and respiratory morbidity in Colombian schoolchildren	2010	Height was measured to the nearest 1 mm with wall-mounted portable SECA 202 stadiometers
18	Estado nutricional y hábitos alimentarios en Indígenas Embera de colombia	2010	To measure length in children under 24 months, an infantometer designed especially for this study was used, with a capacity of 1 m and a sensitivity of 0.1 cm. Height in children 24 months and older was measured in centimeters using a SECA brand portable stadiometer (SECA Deutschland, Germany) with a capacity of 2 m and a sensitivity of 0.1 cm.
19	Propuesta metodológica para la comparación de mediciones antropométricas entre una población base y una población objeto: una aplicación entre población colombiana y estadounidense	2011	N.A
20	Factors associated with stunted growth in children below 11 years of age in Antioquia, Colombia, 2004	2011	Height was measured with an assembled and portable stadiometer (SECA 213), with a capacity of 210 cm and a sensitivity of 0.1 cm.
21	Variabilidad del peso, la estatura y el índice de masa corporal según desarrollo puberal y tipo de colegio en adolescentes de Medellín, Colombia	2012	A stadiometer brand Jandac 220 cm long and 0.1-cm precision was used.
22	The double burden of malnutrition and its risk factors in school children in Tunja	2012	Height was measured with a Kramer portable stadiometer with base, capacity 1 to 2 m, and the weight with a Detecto medical scale (0–130 kg)
23	Estudio transversal de crecimiento de los escolares bogotanos: valores de estatura, peso e índice de masa corporal de los siete a los dieciocho años	2012	Height was evaluated with a rigid wall height rod from 60 to 210 cm, with a precision range of 0.1 cm, barefoot and without stockings.
24	Socioeconomic inequalities in malnutrition among children and adolescents in Colombia: the role of individual-, household- and community-level characteristics	2013	Colombian Demographic and Health Survey
25	Estado nutricional y determinantes sociales asociados en niños Arhuacos menores de 5 años de edad	2013	N.A
26	The dual burden of malnutrition in Colombia	2014	Colombian Demographic and Health Survey
27	Estado nutricional antropométrico de los niños y adolescentes de 17 escuelas del área rural del municipio de La Mesa, Cundinamarca, Colombia, 2012	2014	A height rod of the SECA 213 type was used, with a maximum capacity of 2 m and a precision of 1 mm. Everyone was asked to remove their shoes and accessories on their heads
28	Determinantes socioeconómicos, inseguridad alimentaria y desnutrición crónica en población desplazada de primera infancia, Pereira, Colombia	2014	N.A
29	Valoración del estado nutricional de la población escolar del municipio de Argelia, Colombia	2014	A Kramer adult tape measure was used
30	Determinantes sociodemográficos de la nutrición infantil en Colombia	2015	Colombian Demographic and Health Survey
31	The nutrition transition in Colombia over a decade: A novel household classification system of anthropometric measures	2015	Colombian Demographic and Health Survey
32	Colombian reference growth curves for height, weight, body mass index and head circumference	2016	Height was measured with the legs and heels extended against a movable table and, subsequently, in a straight standing position using a manual stadiometer.
33	Características nutricionales de los escolares afrodescendientes de la costa pacífica Colombiana	2016	Training about taking proper measurements of weight and height was given to technicians by an expert in nutrition.
34	Nivel y estado nutricional en niños y adolescentes de Bogotá, Colombia.	2016	Height was measured with a SECA 206^®^ portable stadiometer (Hamburg, Germany), range 0–220 cm with 1-mm precision.
35	Estado nutricional y determinantes sociales en niños entre 0 y 5 años de la comunidad de Yunguillo y de Red Unidos, Mocoa—Colombia	2016	For those over two years of age, the measurement was be made standing up (vertical position) taken with a height rod.
36	Inequalities in the reduction of child stunting over time in Latin America: evidence from the DHS 2000–2010	2018	Colombian Demographic and Health Survey
37	Peso, estatura e índice de masa corporal de niños y adolescentes de moderada altitud de Colombia	2018	It was measured using a portable stadiometer (SECA Gmbh & Co. KG, Hamburg, Germany), with a precision of 0.1 mm.
38	Un estudio sobre el crecimiento, estado nutricional y composición corporal en menores de quince años de Salgar, Puerto Colombia: variabilidad y determinantes sociales	2020	Height was measured with a SECA^®^ 120 stadiometer (accuracy +/− 1 mm),

**Table A3:** Journals of the reviewed articles.

Revista Médica de Risaralda
Acta Pædiatrica
American Society for Nutrition
Archives of Public Health
Archivos Argentinos de Pediatría
Archivos Latinoamericanos de Nutrición
Biomédica
BulIetin of PAHO
Colombia Médica
Coyuntura Económica—Fedesarrollo Coyuntura Social-Fedesarrollo
Ecology of Food and Nutrition,
Food and Nutrition Bulletin,
Nutrición Hospitalaria
Oxford Development Studies
Perspectivas en Nutrición Humana
Public Health Nutrition
Revista CES Odontología
Revista chilena de nutrición
Revista de la Facultad de Medicina de la Universidad Nacional de Colombia
Revista de la sociedad colombiana de endocrinología
Revista de Salud Pública de la Universidad nacional
Revista Española de Salud Pública
Revista Panamericana de Salud Pública
Revista Salud Bosque
Revista Universidad y Salud
Salud Uninorte
